# Progress in Research on the Role of FGF in the Formation and Treatment of Corneal Neovascularization

**DOI:** 10.3389/fphar.2020.00111

**Published:** 2020-02-25

**Authors:** Mengji Chen, Licheng Bao, Mengying Zhao, Jiarong Cao, Haihua Zheng

**Affiliations:** Department of Ophthalmology, The Second Affiliated Hospital and Yuying Children’s Hospital of Wenzhou Medical University, Wenzhou, China

**Keywords:** corneal neovascularization, fibroblast growth factor, development, treatment, drug delivery

## Abstract

Corneal neovascularization (CNV) is a sight-threatening disease usually associated with inflammatory, infectious, degenerative, and traumatic disorders of the ocular surface. Fibroblast growth factor (FGF) family members play an important role in angiogenesis to induce corneal neovascularization, which significantly affects the differentiation, proliferation, metastasis, and chemotaxis of vascular endothelial cells. Both acidic fibroblast growth factor (aFGF) and basic fibroblast growth factor (bFGF) demonstrate positive staining in capillaries and induce corneal stromal cells. The anabolism of endothelial cells is induced by bFGF in corneal neovascularization. FGFs exert their effects *via* specific binding to cell surface-expressed specific receptors. We believe that both anti-FGF antibodies and anti-FGF receptor antibodies represent new directions for the treatment of CNV. Similar to anti-vascular endothelial growth factor antibodies, subconjunctival injection and eye drops can be considered effective forms of drug delivery.

## Corneal Neovascularization

The incidence rate of corneal neovascularization (CNV), a sight-threatening condition, is approximately 1.4 million patients per year (Bonini, 2016). It is usually associated with inflammatory, traumatic, or infectious disorders of the ocular surface. Corneal neovascularization can cause vision damage and even blindness, the rate of which is as high as 57.4% (Chang et al., 2001). The cornea needs to be transparent to allow the passage of light into the retina. When CNV occurs, abnormal blood vessels directly block light, indirectly diffract light as canals for inflammatory cells, and damage the structure of the cornea by depositing lipids and proteins into the corneal stroma. Various mechanisms of corneal neovascularization have been studied. A balance exists between angiogenic factors (such as fibroblast growth factor and vascular endothelial growth factor) and anti-angiogenic factors (such as angiostatin, endostatin, and pigment epithelium-derived factor) in the cornea (Senturk et al., 2016). An imbalance leads to CNV, which can be divided into superficial neovascularization and deep stromal neovascularization. In the normal cornea, heparan sulfate prevents the release of potent angiogenic cytokines, such as fibroblast growth factor (FGF) and vascular endothelial growth factor (VEGF). However, under stimulation, cytokines may be released, causing an imbalance (Gurung et al., 2018).

## FGF

### FGF

Fibroblast growth factor participates in cell proliferation, migration, and tissue repair in adults (Itoh and Ornitz, 2004). FGFs can stimulate many cell types, including endothelial cells and nerve cells. The FGF family comprises 22 members (FGF1-23, except FGF15 because mouse FGF15 is the orthologue of human and chick FGF19) (Beenken and Mohammadi, 2009). Many of these FGFs, particularly FGF-1 (acidic FGF) and FGF-2 (basic FGF), have been shown to influence angiogenesis in several tissues *in vivo* (**Table 1**) by acting on endothelial cells. Different factors affect FGFs in different tissues. FGFs are produced by endothelial cells and are stored in the extracellular matrix. They show a high affinity for heparin (Klagsbrun, 1990).

**Table 1:** Fibroblast growth factors (FGFs) associated with angiogenesis.

FGFs	Pathway/influencing factor	Related tissue/disease
FGF-1	S156C-TIMP3 mutation	Choroid (Loren et al., 2009; de Oliveira Dias et al., 2011)
	P53	Inflammed tissue (Zhou et al., 2009)
	Erk and MMP-7	Colon cancer (Jin et al., 2016)
	S100A13	Endometriosis (Francavilla et al., 2013)
FGF-2	VEGF	Corneal, choroid, and retina (Senturk et al., 2016)
	NDY1/KDM2B-miR101-EZH2	Tumor tissue (Akpek et al., 2004)
	Interleukin-1β	Chondrocytes (Baradaran-Rafii et al., 2019)
	AKT/MMP-2	Human umbilical vein (Mimura et al., 2011)
FGF-3	Erk and MMP-7	Tumor tissue (Nor et al., 2001)
FGF-8	Co-expression of VEGF	Prostate cancer (Stevenson et al., 2012)
FGF-9	VEGF-A	Bone (Koolwijk et al., 1996)
FGF-18	Wnt/β-catenin	Hepatocellular carcinoma (Zheng et al., 2001)
FGF-21	Dynamin-2 and Rab5	Kidney (Kim et al., 2013)

FGFs activate transmembrane tyrosine kinases and their coupled intracellular signalling pathways to stimulate biological activities and transduce external signals through the PI3K, MAPK, and phospholipase Cγ (PLCγ) pathways (Klagsbrun, 1990; Zhou et al., 2009; Francavilla et al., 2013; Huang et al., 2016). A dual-receptor system comprising a family of four receptor tyrosine kinases (FGFRs) and heparan sulfate proteoglycans (HSPGs) mediates the stimulation of cellular metabolism by FGFs. Thus, many of the FGFR isoforms bind several FGFs. HSPG receptors may provide additional specificity (Klagsbrun, 1990). ERK1/2, JNK1/2, and p38 alpha/beta are three main MAPK subgroups (Zhang et al., 2009). Phospholipase C can receive the signal from FGFRs and then activate PLCγ1 to induce cell proliferation and migration in vascular smooth muscle cells by cleaving the phospholipid phosphatidylinositol 4,5-bisphosphate into diacylglycerol and inositol 1,4,5-triphosphate (Michel, 1998). Studies have suggested that heparan may modulate the activity of FGFs (Ornitz, 2000). Additionally, studies have suggested that membrane-type 1 matrix metalloproteinase (MT1-MMP) plays an important role in modulating FGF-mediated signal pathways (Zhou et al., 2000). It controls FGF signalling by reducing the amount of FGF bound to the cell surface, increasing the proteolysis of FGFs and the downregulation of FGFR-1 and -4 (Tassone et al., 2015).

### FGFs in the Eye

mRNAs encoding basic FGF are produced by corneal epithelial cells, stromal fibroblasts, and corneal endothelial cells (Oladipupo et al., 2014). FGF-2 may have different functions in the three primary cell types of the cornea. Studies have shown that in corneal epithelial cells, FGF-2 stimulates the proliferation of corneal epithelial cells and increases the rate of epithelial wound healing through autocrine and paracrine effects (Kanayama et al., 2007). Rajesh et al. showed that FGF-2 promotes corneal stromal wound healing by increasing cellular proliferation (stimulated by TGF-beta *via* paracrine effects) (Moioli et al., 2006) and cellular motility (significant enhancement of stromal fibroblast motility by 100 ng/ml FGF-2 in animal experiments) (Rao et al., 1992). Cdc42 activation, Rho inactivation, and the phosphatidylinositol 3-kinase pathway in corneal endothelial cells (CECs) are essential for FGF-2-induced wound healing (Lee and Kay, 2006), and endothelial mesenchymal transformation can be mediated by FGFs in CECs. Additionally, FGFs can mediate the proliferation and regeneration of the lens (FGF-1 and FGF-2) (Zhu et al., 2012) and retinal cells (FGF-2, FGF-5, and FGF-9) through different signalling pathways (Gong et al., 2014). FGF-9, FGF-21, and FGF-23 are also expressed in choroidal endothelial cells and affect choroid plexus epithelial cell behaviour (Loren et al., 2009; de Oliveira Dias et al., 2011).

FGFs also play an important role in early mammalian eye development. The neuroepithelium of the optic vesicle separates into NR and RPE domains in a manner mediated by extrinsic factors that emanate from the surface ectoderm, for which fibroblast growth factors are prime candidates (Bassnett and Sikic, 2017).

## FGFs and Related Corneal Diseases

Many FGFs affect angiogenesis, but FGF-2 is the most common isoform to be detected in the eye and to cause CNV in different eye diseases. One of these diseases is ocular chemical burn. We focused on alkali burns because of their severity. CNV can be caused by severe corneal alkali burn (Nominato et al., 2018), in which deep corneal stroma or full-thickness corneal injury is involved. In this situation, angiogenic factors play an important role in CNV. For example, experimental data have shown that on the second day after alkaline burn, b-FGF is obviously expressed in the corneal epithelium, substantia propria layer, and endothelium (Xiao et al., 2012). Additionally, studies have shown that the upregulation of related genes (microRNA-296) after alkali burn is positively correlated with the expression of related FGF isoforms (FGF-23). FGF-23 may inﬂuence corneal inﬂammatory responses by participating in cytokine-cytokine receptor interaction pathways (Hayashi et al., 1996).

Keratitis is another important cause of corneal neovascularisation (Soiberman et al., 2017). In the initial stage of infection with herpes simplex virus type 1 (HSV-1), both virus and immune cells are present in the cornea. Meanwhile, various cytokines and growth factors (FGF-2 and Ang-2), which also permeate the cornea, lead to further inflammation. The origin of FGF-2 may not fibroblasts (keratocytes), epithelium, endothelium, blood endothelial cells, and lymphatic endothelial cells rather than leukocytes (Xu et al., 2019). In the late stage, immune cells and growth factors continue to be effective while the virus is removed from the cornea. Furthermore, FGF-2 mediates the expression of other cytokines (such as VEGF-A, IL-6, and Ang-2), which are crucial for HSV-1-induced corneal neovascularization (Klagsbrun, 1990).

Patients who undergo corneal transplantation can develop corneal neovascularization. Particularly, after high-risk keratoplasty, intense CNV outgrowth is a common phenomenon in the early postoperative period (Kelly et al., 2011). Corneal neovascularization can lead to an increased risk of graft rejection caused by an imbalance between angiogenic factors and anti-angiogenic factors. Additionally, FGF is an important angiogenic factor. Presently, no study has shown how FGFs cause CNV after corneal transplantation and which FGF isoform is involved, but this phenomenon warrants further research.

## Role of FGFs in CNV Formation

**Figure 1:**
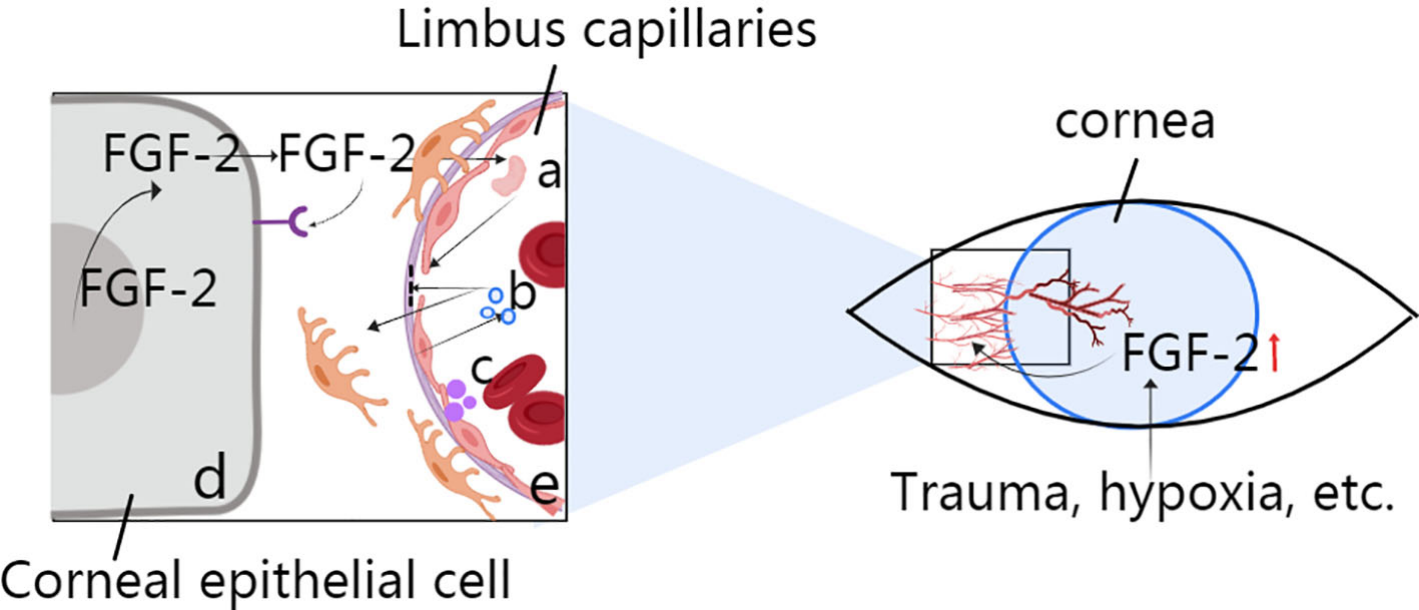
Role of FGF-2 in corneal angiogenesis. **(A)** Activation for the destruction of endothelial cell junctions *via* MMP secretion. **(B)** Disruption of the basal membrane and pericytes *via* ANG2. **(C)** Secretion from vascular endothelial cells to induce blood vessel destabilization. **(D)** Corneal epithelial cell production through autocrine or nuclear actions during events such as trauma and hypoxia. **(E)** Limbus capillaries: activation of Src family kinases.

Angiogenesis is the process by which new blood vessels grow by sprouting from established blood vessels (Carmeliet and Jain, 2011). In the cornea, these existing blood vessels can be part of the vascular plexus around the limbus of the anterior ciliary artery. Corneal angiogenesis occurs because of the release of proangiogenic factors, such as FGF-2, VEGF, and several other chemokines, from hypoxic or inflammatory cells. The binding of angiogenic factors to corresponding receptors on vascular endothelial cells leads to many events, including the following: (a) injury to endothelial cell junctions through the activation of non-receptor Src family kinases and increased expression of integrins; (b) the promotion of endothelial cell proliferation by mitogen-activated protein kinase and phosphoinositide 3' kinase; (c) the secretion of metalloproteinases (MMPs) by endothelial cells to promote basal membrane disruption and pericyte detachment; and (d) blood vessel destabilization caused by the release of angiopoietin 2 (ANG 2) from endothelial cell granules (**Figure 1**). Additionally, murine tissue inhibitor of metalloproteinase-4 (TIMP-4) expression in the cornea may play a role in regulating extracellular matrix remodelling associated with corneal wound healing and angiogenesis; however, the mechanism is unclear.

Vascular endothelial cells are stimulated directly by FGFs from angiogenic tissues. FGF-producing cells also release FGFs through autocrine or nuclear actions (**Figure 1**), and secondary angiogenic factors then act on the vasculature. The vascular endothelium can induce the secretion of secondary regulatory molecules in the same way, and such secretion can also be influenced by other angiogenic factors, such as VEGF. The specific role of FGFs depends on the environment in which they are located (Beenken and Mohammadi, 2009).

## Progress in FGF Research for CNV Treatment

### Current Effective Treatments for CNV

Presently, laser treatment, drug treatment, and surgical treatment are available to treat corneal neovascularization. Drugs used for treatment include glucocorticoids, immunosuppressive agents, and various vascular growth factor inhibitors. While the topical administration of steroidal anti-inflammatory drugs is the first-line treatment for corneal neovascularization, many side effects occur during long-term application (Mukwaya et al., 2019). Laser treatment is less effective for the dense mesh type of CNV. Corneal transplant surgery may also cause various complications, including HSV-1 infection of the cornea.

A novel therapy targeting angiogenic cytokines may have therapeutic potential for clinical use in the future, and it has been shown to inhibit corneal vascularization effectively in animal models (Klagsbrun, 1990). Repeated subconjunctival injections of Avastin^®^ (the trade name of bevacizumab; 1.25 mg/0.05 ml) and topical cyclosporin-A drops appear to be safe and effective in treating aggressive corneal vascularization (Akpek et al., 2004; Stevenson et al., 2012). Subconjunctival bevacizumab injections were shown to be effective and safe in reducing corneal neovascularization within the first 4 months (Jacobs et al., 2009). Recent research has shown that bevacizumab can also be administered by corneal intrastromal injection for the inhibition of intrastromal vascularization after deep anterior lamellar keratoplasty (Sun et al., 2019). Additionally, there are few corneal epithelial side effects when bevacizumab eye drops are used to treat corneal neovascularization (Baradaran-Rafii et al., 2019). Anti-angiogenic effects and anti-fibrotic effects for the maintenance of corneal transparency have also been observed after the use of bevacizumab eye drops (25 mg/ml) for corneal burn (Koenig et al., 2012). However, only a few related studies using eye drops exist, and further verification is needed.

### Comparison of Anti-FGF and Anti-VEGF

Anti-VEGF drugs include recombinant full-length VEGF monoclonal antibodies (Avastin), recombinants of VEGF subtype monoclonal antibody fragments (Lucentis), and RNA aptamers (Macugen). The mechanism of these drugs involves binding to VEGF and inhibiting the specific binding of VEGF to its receptor. Recent observations have shown that the soluble pattern recognition receptor long-pentraxin-3 binds FGF2 with high affinity and specificity and thus acts as an FGF2 antagonist (Presta et al., 2018). The novel RNA aptamer (a short single-stranded nucleic acid molecule) APT-F2, which is specific for human FGF2, was also recently discovered as an anti-FGF drug (Jin et al., 2016), but it has not been applied in anti-angiogenic studies. More types of anti-FGF drugs are yet to be discovered. The mechanism of anti-FGF drugs is similar to that of anti-VEGF drugs.

Studies have demonstrated that bFGF expression induces VEGF, MT1-MMP, and CD31 in the experimental mouse cornea (Nor et al., 2001; Mimura et al., 2011). Therefore, FGF treatment may be more comprehensive. Researchers believe that angiogenesis induced by basic fibroblast growth factor (bFGF) is immune to anti-VEGF/VEGFR (vascular endothelial growth factor/receptor) therapy (Koolwijk et al., 1996). Furthermore, patients may develop tolerance to anti-VEGF drugs. Experimental evidence suggests that FGF2-targeted drugs might provide cooperative effects with anti-VEGF monoclonal antibodies for the treatment of angiogenesis-related diseases. Another important advantage of anti-FGF drugs is that they may be beneficial for the recovery of vision. Researchers established a model of anti-FGF-2 antibody-treated mouse corneal neovascularization and then evaluated the corneal sensitivity and visual acuity of these mice to assess their functional vision. Time-course experiments revealed partial recovery of visual acuity in mice treated with an anti-FGF-2 antibody compared with control mice (Zheng et al., 2001). This finding supports anti-VEGF therapy for corneal neovascularization, but further experimental confirmation is needed.

### FGF as a Drug Source and the Administration Route

Several anti-FGF2 monoclonal antibodies (mAbs) that can neutralize the activities of FGF2 *in vitro* and *in vivo* have been identified (Jin et al., 2016). However, to our knowledge, no anti-FGF2 mAb has entered clinical trials. Because of the similarity between VEGF and FGF antagonists, we can presume the likely effect of FGF antagonists in treating CNV based on the effects of anti-VEGF drugs.

A previous study demonstrated that the subconjunctival injection of anti-VEGF drugs (1.25 mg/0.05 ml bevacizumab) significantly reduces inflammation and fibroblast activity to inhibit corneal neovascularization in the cornea of a rat model of alkali burn. Rats in the test group were treated with a subconjunctival injection (Kim et al., 2013). Another researcher advocated combining subconjunctival (1.25 mg/0.05 ml) and intracorneal injection (1.25 mg/0.05 ml). They confirmed that combining subconjunctival and intracorneal injection did not damage corneal endothelial cells (Lichtinger et al., 2014). No clinical case featuring the administration of anti-FGF drugs through subconjunctival and intracorneal routes for the treatment of CNV exists, but we can refer to these modes of administration. Therefore, the drug concentration and side effects remain unclear, and further research is needed.

Recently, research on new eye drops has progressed. Considering the effects of anti-VEGF drugs, in the future, Avastin eye drops may be shown to exhibit the same anti-VEGF effects with fewer complications than intravitreal use (Kim et al., 2013). Bevacizumab eye drops seem to inhibit corneal neovascularization without inducing obvious corneal epithelial side effects. Bevacizumab eye drops are prepared by adding 0.9% physiological saline to the drug at a concentration of 5 mg/ml. The minimum storage temperature of the eye drops is – 20°C (before opening), the maximum storage temperature is 4°C (after opening), and the maximum storage time is 14 days; after opening, they needs to be used within 1 day (Bock et al., 2008). Therefore, we believe that preparations of anti-FGF drugs as eye drops can be applied for the clinical treatment of corneal neovascularization. The effects of the FGF antagonist tecogalan sodium against corneal neovascularization were tested in animal experiments, and tecogalan sodium demonstrated dose-dependent antiangiogenic activity. The inhibitory effect of 100 ng terconazole sodium was weakly, and the effect of 250 ng was marked, indicating that corneal neovascularization induced by bFGF can be inhibited by the topical instillation of tecogalan sodium (Murata et al., 1995). However, no data regarding the recommended dose of other anti-FGF drugs for the treatment of CNV are available. It is possible that we need to start with a low dose and simultaneously test the corneal toxicity of the drug.

## Summary

Corneal neovascularization is a pathological change in the cornea that blocks light, leads to inflammation and edema, and causes corneal scarring in severe cases. Ultimately, it leads to a serious decline in vision. When the balance between angiogenic and antiangiogenic factors shifts towards the former, corneal neovascularization occurs. FGF, particularly bFGF, plays a very important role in corneal neovascularization due to various factors. However, its exact role and mechanism require further research. Therefore, anti-FGF drugs can be used as new candidates for treating corneal neovascularization. Presently, steroidal anti-inflammatory drugs are the first-line therapy for corneal neovascularization. New types of drugs with fewer side effects need to be developed; anti-VEGF drugs are one of the candidates. Compared with anti-VEGF drugs, anti-FGF drugs have advantages. The discovery of additional FGF antagonists and the route of administration of anti-FGF drugs will become a new research direction. The authors believe that the subconjunctival and corneal stroma injection of anti-FGF drugs and anti-FGF eye drops will provide new strategies to treat corneal neovascularization.

## Author Contributions

MC and HZ participated in drafting the manuscript. LB, MZ, and JC provided technical assistance. MC and HZ revised the manuscript. HZ supervised the project and provided financial support. MC and HZ wrote the main part of the paper. All of the authors read and approved the final manuscript.

## Conflict of Interest

The authors declare that the research was conducted in the absence of any commercial or financial relationships that could be construed as a potential conflict of interest.
